# Supplementary material for: A Robust Phenotypic High-Throughput Antiviral Assay for the Discovery of Rabies Virus Inhibitors
Source: Viruses. 2023 Nov 23;15(12):2292. doi: 10.3390/v15122292 (PMC10747594; doi:10.3390/v15122292)
Supplement: Supplementary file 1 [file viruses-15-02292-s001.zip › viruses-2716509-supplementary.pdf]

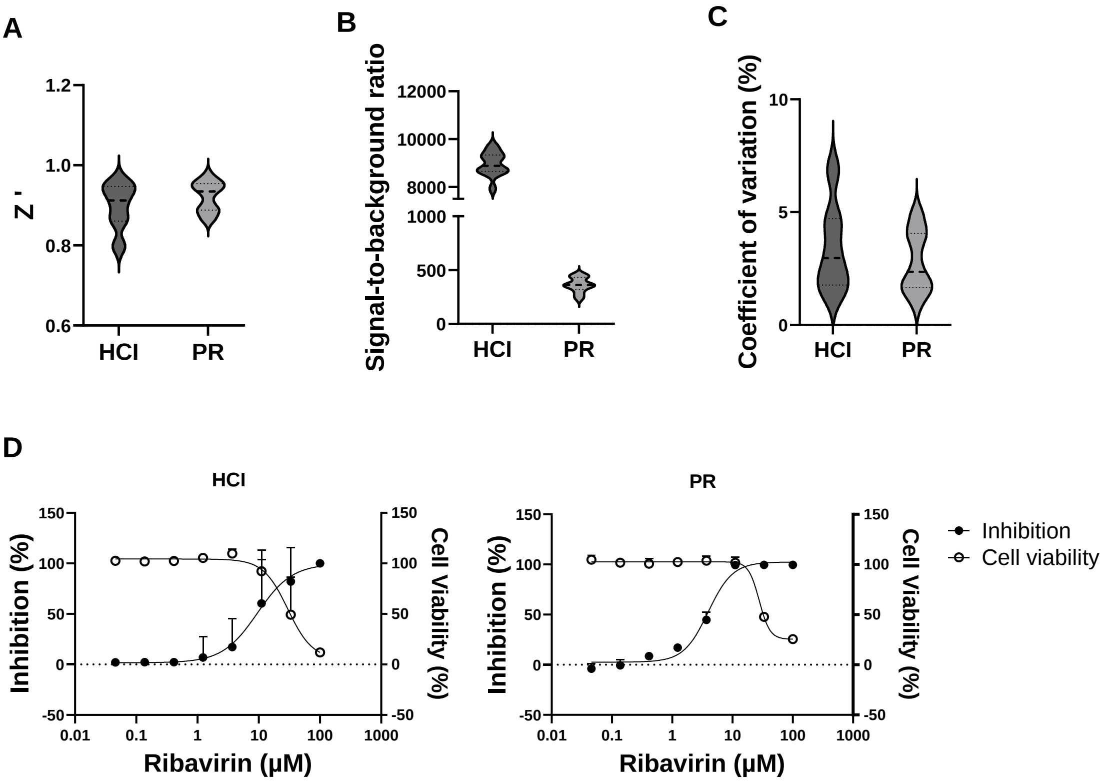


**Figure S1.** Antiviral activity comparison of HCI and PR assay. Z′ (A), S/B (B) and CV% (C) of HCI (used for HTS) and plate-reader (PR = whole-well fluorescence – used for hit confirmation) antiviral assays. The average Z′ factors are 0.90 in HCI and 0.92 in PR; signal-to-background ratio are 8960 in HCI and 362 in PR; percentage of coefficient of variation of infected control are 3.4% in HCI and 2.7% in PR. The violin plot graphs show data from 13 plates. (D) Anti-RABV activity of ribavirin in BHK-21 cells in HCI and PR assay. Serial dilutions of ribavirin were added together with mCherry-RABV (MOI 0.019 in HCI assay, MOI 0.01 in PR assay) to BHK-21 cells. Antiviral activity and cell viability were determined by HCI analysis for 384-well assay, or by using a standard plate reader (PR) for 96-well assay. Fitting the dose-response curves results in the calculation of $EC_{50}$ of ribavirin of 9.5 µM and 3.9 µM, and $CC_{50}$ of 30 µM and 28 µM, respectively. Averages and standard deviations of at least 3 independent experiments are presented.

**Table S1.** Effect of selected molecules on *in vitro* RABV replication.

| Compounds | Screening antiviral activity at 10 µM | | Dose response confirmation | | |
|---|---|---|---|---|---|
| | % infection | % viability | $EC_{50}$ (µM) [a] | $CC_{50}$ (µM) [b] | SI [c] |
| Cyproheptadine | 21 | 78 | 5.6 ± 0.95 | 39 ± 13 | 6.9 |
| Levamlodipine | 5.3 | 60 | 7.9 ± 3.1 | 24 ± 2.0 | 3.1 |
| Dasatinib | 7.0 | 65 | 2.0 ± 2.6 | 5.8 ± 4.8 | 2.9 |
| Salinomycin | 20 | 63 | <0.080 | 3.0 ± 0.8 | >38 |
| Ribavirin [d] | 40 | 92 | 5.1 ± 1.6 | 36 ± 18 | 7.0 |

a. Concentration at which present virus infection was reduced by 50%.
b. Concentration at which cell viability was reduced by 50%.
c. Selectivity index (ratio of $CC_{50}$ to $EC_{50}$).
d. Included as reference compound.
